# Supplementary material for: Chromothripsis during telomere crisis is independent of NHEJ, and consistent with a replicative origin
Source: Genome Res. 2019 May;29(5):737–49. doi: 10.1101/gr.240705.118 (PMC6499312; doi:10.1101/gr.240705.118)
Supplement: Supplemental Material [file supp_gr.240705.118_Supplemental_file_1.zip › contigs/annotated_contigs/DB53/contig.2.DB53_length_819_mean_cov_14.514041514.docx]

**DB53_length_819_mean_cov_14.514041514**

```
AATCTCCCAGTTTGTGCTATTGATATATCAGGC|CATTCTTACTTTCTGTCTCCTCTGCCTGGAACAAGCTGCCATCATATCTTCATAG
                                  >chr8:77845207-77845650 + E=1e-254
AGCTGGATCCTTGTTGTTTGTTTCAACTCAAATGTCACCTCCTTAGAGAGAGGCCTTCGAGTCTATCTACTATACCATATTCTCATGCC

TACTCAATCTATATCATATTTTCCTAATTTTATGGACTTATCACTAGCAAAAATGGCTATGTTCATTAATTTGTTAATTGCTTATTGTT

TCTTCCTCCCTTGGGAATGCAAGCTCCATCAGAGCACTTACATTCTTACACTAAAATACATCTAGTGCCCAACATCCTACCTAGGTGGG

TTTTAATAAACATATATTGAATGAAATAAGAGATTTCACTTCAAAGCAAGGTTCTACTTTAAAAAAAAACTTAACACTATGGCTCCTAC

TCTGACATGCGGTTAAATATTTACTC|TTTGCA|GTTTTTACCTAACTAGAGTCAAGGATCCCACAATTCAGAGAGTTAATAGAGGCAA
                           >chr8:85360563-85360912 - E=4e-198
TACTCCTTGAAGTTAGTTACTTACTGAAGAAAAAGCGTGTAGCAGTGCTTTGTTAAATGACAACAATTCTATCAAGTGAATAATCCTGT

ATGAAGGTTTTTCATCACCTCTCTTCATAAGTGGTGCACTATATGGGGGCGGGGGCAGAAATGCAATTGAGGCAAGAGAATAGGTATCT

TTTTAAAAACTAACATTATTTTTAACGAGTTATCAAATTGAAATTGTCCATGAGTTGCACATAATTTCAAACGTCTCAACAACTAAAAT

TGGAGCATGCTTAAACTCTCT
```
